# Association study between autistic-like traits and polymorphisms in the autism candidate regions *RELN, CNTNAP2, SHANK3,* and *CDH9/10*

**DOI:** 10.1186/2040-2392-5-55

**Published:** 2014-12-16

**Authors:** Lina Jonsson, Anna Zettergren, Erik Pettersson, Daniel Hovey, Henrik Anckarsäter, Lars Westberg, Paul Lichtenstein, Sebastian Lundström, Jonas Melke

**Affiliations:** Department of Pharmacology, Institute of Neuroscience and Physiology at the Sahlgrenska Academy, University of Gothenburg, POB 431, SE 405 30 Gothenburg, Sweden; Department of Medical Epidemiology and Biostatistics, Karolinska Institutet, Stockholm, Sweden; Department of Forensic Psychiatry, Institute of Neuroscience and Physiology at the Sahlgrenska Academy, University of Gothenburg, Gothenburg, Sweden; Swedish Prison and probation service, R&D unit, Gothenburg, Sweden; Gillberg Neuropsychiatry Centre, Institute of Neuroscience and Physiology at the Sahlgrenska Academy, University of Gothenburg, Gothenburg, Sweden

**Keywords:** Autistic-like traits, Autism spectrum disorder, *CNTNAP2*, *RELN*, *rs4307059*, *SHANK3*, *A-TAC*, *CATSS*

## Abstract

**Background:**

Autistic-like traits (ALTs) are continuously distributed in the general population, with the autism spectrum disorder (ASD) at the upper extreme end. A genetic overlap has been shown between ALTs and ASD, indicating that common variation in ASD candidate genes may also influence ALTs. In our study, we have investigated the SNP rs4307059 that has been associated with both ALTs and ASD. In addition, we genotyped polymorphisms in a selection of genes involved in synaptic functioning, *that is, SHANK3*, *RELN,* and *CNTNAP2,* which repeatedly have been associated with ASD. The possible associations of these polymorphisms with ALTs, as well as genetic factors for neurodevelopmental problems (NDPs), were investigated in a large cohort from the general population: The Child and Adolescent Twin Study in Sweden. For analyses of ALTs and NDPs, 12,319 subjects (including 2,268 monozygotic (MZ) and 3,805 dizygotic (DZ) twin pairs) and 8,671 subjects (including 2,243 MZ and 2,044 DZ twin pairs), respectively, were included in the analyses.

**Findings:**

We could not replicate the previous association between rs4307059 and social communication impairment. Moreover, common variations in *CNTNAP2* (rs7794745 and rs2710102), *RELN* (rs362691), and *SHANK3* (rs9616915) were not significantly associated with ALTs in our study.

**Conclusions:**

Our results do not suggest that the investigated genes, which previously has been found associated with ASD diagnosis, have any major influence on ALTs in children from the general population.

**Electronic supplementary material:**

The online version of this article (doi:10.1186/2040-2392-5-55) contains supplementary material, which is available to authorized users.

## Findings

### Background

Autism spectrum disorder (ASD) is a heterogeneous group of neurodevelopmental disorders that are characterized by impairments in social interaction restricted and repetitive behavior and communication impairments. Furthermore, there is a large co-morbidity between ASD and other neurodevelopmental disorders, such as Attention Deficit/Hyperactive Disorder (ADHD) [1] and a shared genetic susceptibility has been suggested for neurodevelopmental disorders [2].

The phenotypic heterogeneity within ASD is probably the major explanation for the inconclusive results from genetic studies of ASD. Indeed, the majority of genes and biological pathways clearly implicated in ASD have been identified in studies of rare variation [3], whereas studies of common polymorphisms, both in large, genome-wide association studies (GWAS) [4–6] and smaller hypothesis driven studies have yielded inconclusive results [3]. Another approach to investigate the role of common variants in ASD is to assess milder, non-pathological phenotypes related to ASD, such as autistic-like traits (ALTs), that are dimensionally distributed in the general population [7]. Theoretically, common variations would have a larger impact for ALTs than for severe ASD [3], and this approach also allows for analyses of the genetics for different aspects of the autistic phenotype separately [8]. Indeed, it has been shown that ALTs and clinical ASD are etiologically related [7] and one example of a single nucleotide polymorphism (SNP) that has been associated both with ASD [5] and social interaction impairment is a SNP (rs4307059) between the genes Cadherin 9 and 10 (*CDH9* and *CDH10*) [9]. Also, a SNP (rs2710102) in the Contactin associated protein-like 2 *(CNTNAP2)* has been shown to affect language development in the general population [10] and age at first word in children with ASD [11].

Genetically determined abnormalities in neurodevelopment and synaptic functioning are increasingly recognized as a cause of ASD. Although these findings primarily are based on rare mutations identified in ASD families [12–14], there is evidence for an influence of also common polymorphisms in ASD [3]. In the present study we initially evaluated the association between common variation in three genes involved in neurodevelopment and/or synaptic function, that is, *CNTNAP2*, SH3, and multiple ankyrin repeat domains 3 (*SHANK3)* and Reelin (*RELN*)*,* and continuous measures of ALTs. Second, we investigated the possible influence of rs4307059 (between *CDH9* and *CDH10*) and rs2710102 (*CNTNAP2*) on both ALTs and ASD. Finally, we explored the influence of the five SNPs on neurodevelopmental problems (NDPs). Thus, the purpose of our study was to expand and partly replicate previous findings regarding these SNPs in autism related phenotypes.

### Subjects and methods

#### Participants and measurements

The subjects in our study are part of The Child and Adolescent Twin Study in Sweden (CATSS) that is focusing on neurodevelopmental problems in children [15]. Our sample from the CATSS included 2,301 monozygotic (MZ) twin pairs, 3,870 dizygotic (DZ) twin pairs, and 84 subjects included without their co-twin. From this sample, we have excluded 107 subjects due to documented brain damage or a known genetic syndrome; a total of 12,319 subjects were included in our analyses of ALTs. For analysis of NDPs a total of 8,671 subjects were included: 2,243 MZ and 2,044 DZ twin pairs, and 97 subjects without their co-twin. The CATSS study has ethical approval from the Karolinska Institutet Ethical Review Board, and informed consent was obtained from the participants.

The parental questionnaire Autism-Tics, ADHD, and other Co-morbidities inventory (A-TAC) was answered by the parents in connection to their twins’ 9th or 12th birthday [16, 17]; 70% are 9 years old (N = 8,623) and 30% are 12 years old (N = 3,696) at the time of testing. The A-TAC consists of 96 questions, of which 17 measure ALTs: six questions correspond to the language impairment, six to the social interaction impairment, and five to restricted and repetitive behavior. The A-TAC score can also be used as a proxy for a clinical ASD diagnosis if the children score higher than 8.5 points on the scale (N = 90 in our sample) [17]. Furthermore, 53 items in the A-TAC have been used to identify a general genetic factor, that is, a factor that consists of all neurodevelopmental problems (NDPs) as well as three genetic subfactors (tics and autism, hyperactivity, and learning problems) that were independent of the general genetic factor and primarily identified by a smaller number of specific symptoms [18]. These four factors were based on over 6,500 twin pairs and were designed to have 100% heritability by fixing the correlations across twin pairs at their expected genetic pedigree (that is, at r = 1.0 for MZ and at r = 0.50 for DZ twins). Since the calculations are based on a heritability estimate that is twice the difference between MZ and DZ twins, the factors end up having 100% heritability. Please note that the 100% heritability is a statistical routine to maximize factor loading, rather than an actual estimate of the heritability of these traits. By relying on the expected twin correlations, factor analysis identified the specific set of weights for the items that were perfectly heritable. Because one factor had substantial and positive weights on all 53 items, it was considered general, that is, all symptoms partly shared the same genetic origin. Given that these factors were entirely genetic in origin, their associations with specific candidate genes may emerge more clearly.

#### Genotyping and statistical analyses

Five single nucleotide polymorphisms (SNPs) in the genes *CNTNAP2* (rs2710102 and rs7794745), *RELN* (rs362691), *SHANK3* (rs9616915), and rs4307059 between *CDH9* and *CDH10* were genotyped. DNA was extracted from saliva using OraGene DNA Self-collection Kit (DNA Genotek Inc., Ottawa, Canada) and the SNPs were genotyped using the Kompetitive Allele Specific PCR (KASP) Genotyping System (LGC Genomics, Herts, UK). All of the SNPs were in Hardy-Weinberg Equilibrium (*P* >0.01) and the genotyping success rate was above 97%.

Statistical association, for ALTs and NDPs, were estimated using linear mixed effect models in the MIXED procedure (PROC MIXED) and for the case–control analyses we used the GLIMMIX procedure of SAS 9.3 (SAS Institute, Inc., Cary, NC, USA). Both of these models allowed us to adjust for the dependent nature of the twin observations, that is, A-TAC scores from all genotyped subjects were included in the analyses. For the MIXED procedure, the genotypes were coded as quantitative measures (0 = major allele homozygote, 1 = heterozygote, and 2 = minor allele homozygote) and effect sizes are presented as the regression coefficients for these analyses. To consider the possible influence of age at testing, all analyses were also performed adjusted for age (see Additional file 1). The Bonferroni corrected *P* value was set to 0.0016 (<0.05/32, adjusted for eight A-TAC measurements and four genetic regions).

We used the Quanto software to analyze which effect sizes (β-value) we would be able to detect with a power of 90% or more using an additive genetic model at a significance level of 0.0016 [19]. The mean values and standard deviation for ALTs, ALT modules, and NDPs were used for the power analyses (N = 12,319). For ALTs we had 90% power to detect the following effect sizes: β-value between 0.08 and 0.09 for all SNPs except for rs36269 (β = 0.12, MAF = 0.13). For the ALT domains (language and social impairment, and restricted and repetitive behavior) the effect size (β-values) ranged between 0.04 and 0.05 and for the genetic factors for NDPs it ranged between 0.03 and 0.06.

## Results and discussion

Our approach was to investigate ALTs and common genetic variations previously implicated in ASD, that is, polymorphisms in *CNTNAP2*, *SHANK3*, *RELN,* and the SNP rs4307059 between *CDH9* and *CDH10*. We could not find any significant association with ALTs for any of the investigated SNPs, however, we did see a nominal significance for the association between the *CNTNAP2* SNP rs7794745 and both social interaction impairment and total ALT scores (Table 1).

**Table 1:** **Association between five SNPs and autistic-like traits**

		Autistic-like traits ^a^							Autistic-like traits domains								
									Restricted & repetitive behavior ^***a***^			Social interaction impairment ^***a***^			Language impairment ^***a***^		
Gene/SNP	MAF	Genotype	N	All	N	Boys	N	Girls	All	Boys	Girls	All	Boys	Girls	All	Boys	Girls
*CDH9/10*																	
rs4307059	0.37 (C)	C/C	1,663	0.68 (1.37)	796	0.86 (1.63)	867	0.50 (1.06)	0.23 (0.57)	0.31 (0.69)	0.16 (0.42)	0.23 (0.57)	0.29 (0.64)	0.18 (0.49)	0.21 (0.52)	0.27 (0.59)	0.16 (0.43)
		T/C	5,564	0.73 (1.44)	2,820	0.91 (1.66)	2,744	0.54 (1.14)	0.23 (0.57)	0.30 (0.66)	0.16 (0.45)	0.25 (0.58)	0.30 (0.64)	0.21 (0.50)	0.24 (0.57)	0.30 (0.65)	0.18 (0.46)
		T/T	4,766	0.67 (1.36)	2,391	0.85 (1.64)	2,375	0.50 (0.98)	0.21 (0.55)	0.28 (0.65)	0.14 (0.40)	0.24 (0.58)	0.29 (0.68)	0.19 (0.45)	0.22 (0.54)	0.27 (0.62)	0.17 (0.44)
*P* value				0.737		0.634		0.724	0.160	0.302	0.162	0.793	0.762	0.669	0.725	0.704	0.357
Effect size (CI 95%)^c^				0.007 (-0.033, 0.047)		0.016 (-0.051, 0.083)		0.007 (-0.033, 0.048)	0.011 (-0.004, 0.027)	0.014 (-0.013, 0.041)	0.011 (-0.005, 0.028)	-0.002 (-0.018, 0.014)	-0.004 (-0.03, 0.022)	0.004 (-0.014, 0.022)	0.003 (-0.019, 0.013)	0.005 (-0.020, 0.030)	-0.008 (-0.026, 0.009)
*CNTNAP2*																	
rs2710102	0.48 (G)	A/A	3,248	0.69 (1.37)	1,618	0.87 (1.63)	1,630	0.52 (1.03)	0.22 (0.57)	0.29 (0.68)	0.15 (0.43)	0.25 (0.57)	0.30 (0.65)	0.20 (0.48)	0.22 (0.53)	0.27 (0.61)	0.17 (0.42)
		A/G	5,976	0.70 (1.41)	2,998	0.88 (1.68)	2,978	0.52 (1.04)	0.22 (0.56)	0.29 (0.66)	0.15 (0.42)	0.24 (0.58)	0.29 (0.66)	0.20 (0.47)	0.23 (0.57)	0.29 (0.65)	0.18 (0.46)
		G/G	2,752	0.73 (1.45)	1,382	0.90 (1.60)	1,370	0.55 (1.24)	0.23 (0.58)	0.31 (0.65)	0.16 (0.47)	0.25 (0.58)	0.29 (0.64)	0.21 (0.51)	0.24 (0.56)	0.30 (0.62)	0.18 (0.49)
*P* value				0.523		0.652		0.727	0.593	0.566	0.880	0.827	0.653	0.766	0.336	0.368	0.542
Effect size (CI 95%)^c^				0.013 (-0.026, 0.052)		0.015 (-0.050, 0.080)		0.007 (-0.033, 0.048)	0.004 (-0.011, 0.020)	0.008 (-0.018, 0.033)	-0.001 (-0.017, 0.015)	-0.002 (-0.018, 0.014)	-0.006 (-0.031, 0.020)	0.003 (-0.015, 0.020)	0.008 (-0.008, 0.023)	0.011 (-0.013, 0.036)	0.006 (-0.012, 0.023)
rs7794745	0.36 (T)	A/A	5,004	0.72 (1.46)	2,475	0.92 (1.73)	2,529	0.53 (1.10)	0.24 (0.60)	0.32 (0.71)	0.16 (0.44)	0.25 (0.59)	0.30 (0.67)	0.19 (0.49)	0.24 (0.57)	0.29 (0.65)	0.19 (0.47)
		T/A	5,533	0.70 (1.41)	2,807	0.87 (1.63)	2,726	0.53 (1.13)	0.22 (0.55)	0.28 (0.63)	0.15 (0.44)	0.26 (0.59)	0.30 (0.66)	0.21 (0.50)	0.23 (0.56)	0.29 (0.64)	0.17 (0.46)
		T/T	1,564	0.67 (1.23)	779	0.82 (1.44)	785	0.51 (0.97)	0.22 (0.52)	0.29 (0.59)	0.15 (0.42)	0.22 (0.50)	0.25 (0.58)	0.20 (0.42)	0.22 (0.51)	0.28 (0.59)	0.16 (0.41)
*P* value				0.102		0.045^b^		0.744	0.126	0.081	0.847	0.309	0.050^b^	0.311	0.182	0.299	0.480
Effect size (CI 95%)^c^				-0.034 (-0.075, 0.007)		-0.069 (-0.136, -0.001)		0.007 (-0.035, 0.049)	-0.013 (-0.029, 0.004)	-0.024 (-0.051, 0.003)	-0.002 (-0.018, 0.015)	-0.009 (-0.025, 0.008)	-0.026 (-0.053, <0.001)	0.009 (-0.009, 0.028)	-0.011 (-0.027, 0.005)	-0.014 (-0.039, 0.012)	-0.007 (-0.025, 0.012)
*RELN*																	
rs362691	0.13 (C)	C/C	211	0.65 (1.34)	107	0.66 (1.11)	104	0.63 (1.54)	0.22 (0.56)	0.25 (0.54)	0.18 (0.58)	0.25 (0.55)	0.24 (0.44)	0.25 (0.65)	0.18 (0.45)	0.17 (0.41)	0.20 (0.49)
		C/G	2,629	0.68 (1.39)	1,267	0.89 (1.69)	1,362	0.49 (1.01)	0.21 (0.56)	0.30 (0.68)	0.14 (0.40)	0.24 (0.60)	0.31 (0.71)	0.18 (0.46)	0.22 (0.54)	0.29 (0.61)	0.16 (0.46)
		G/G	9,139	0.71 (1.40)	4,615	0.88 (1.63)	4,524	0.53 (1.09)	0.22 (0.56)	0.29 (0.66)	0.15 (0.43)	0.25 (0.57)	0.29 (0.64)	0.20 (0.48)	0.23 (0.56)	0.29 (0.64)	0.18 (0.45)
*P* value				0.338		0.629		0.659	0.350	0.739	0.612	0.944	0.835	0.980	0.282	0.306	0.659
Effect size (CI 95%)^c^				-0.028 (-0.086, 0.03)		-0.024 (-0.12, 0.072)		-0.013 (-0.073, 0.046)	-0.011 (-0.034, 0.012)	-0.007 (-0.045, 0.032)	-0.006 (-0.03, 0.017)	-0.001 (-0.024, 0.022)	0.004 (-0.034, 0.042)	<0.001 (-0.026, 0.026)	-0.013 (-0.036, 0.010)	-0.019 (-0.056, 0.018)	-0.006 (-0.032, 0.020)
*SHANK3*																	
rs9616915	0.49 (T)	C/C	3,116	0.74 (1.53)	1,589	0.96 (1.84)	1,527	0.52 (1.07)	0.23 (0.60)	0.32 (0.72)	0.14 (0.43)	0.27 (0.62)	0.33 (0.73)	0.20 (0.48)	0.24 (0.58)	0.31 (0.68)	0.18 (0.44)
		C/T	5,839	0.68 (1.32)	2,906	0.83 (1.51)	2,933	0.53 (1.08)	0.22 (0.56)	0.29 (0.65)	0.16 (0.44)	0.24 (0.54)	0.28 (0.60)	0.20 (0.47)	0.22 (0.52)	0.26 (0.58)	0.18 (0.45)
		T/T	2,927	0.68 (1.38)	1,461	0.88 (1.62)	1,466	0.49 (1.04)	0.21 (0.53)	0.29 (0.62)	0.14 (0.41)	0.24 (0.56)	0.28 (0.64)	0.19 (0.48)	0.24 (0.57)	0.31 (0.67)	0.16 (0.45)
*P* value				0.194		0.425		0.445	0.182	0.274	0.662	0.164	0.184	0.778	0.840	0.637	0.438
Effect size (CI 95%)^c^				-0.026 (-0.064, 0.013)		-0.026 (-0.089, 0.038)		-0.015 (-0.055, 0.024)	-0.010 (-0.026, 0.005)	-0.014 (-0.04, 0.011)	-0.004 (-0.019, 0.012)	-0.011 (-0.027, 0.005)	-0.017 (-0.042, 0.008)	-0.003 (-0.02, 0.015)	-0.002 (-0.017, 0.014)	0.006 (-0.018, 0.030)	-0.007 (-0.024, 0.010)

^a^A-TAC mean values (SD).

^b^Nominal significance, *P* value <0.05.

^c^Effect size presented as the regression coefficient (CI 95%).

A-TAC: Autism-Tics, ADHD, and Other Co-morbidities inventory.

In the *CNTNAP2* gene, the SNP rs7794745 has been associated with autistic disorder [20] while the SNP rs2710102 has mainly been associated with language problems [11]. A study by Whitehouse *et al.* found nominal significance between rs2710102 (*CNTNAP2*) and quantitative measures of early language acquisition in the general population [10], which was not replicated in our study (Table 1).

The SNP rs4307059 in the *CDH 9/10* region has previously been associated with ASD in a GWAS [5], and was replicated in a study by Ma *et al.* [21]. This SNP has also been associated with social communication impairments in a large population [9], however, in line with the results from the two GWAS for autistic traits [22, 23], this could not be replicated in our study (Table 1). However, it should be noted that one of the ALT GWAS included a pooled SNP analysis [23] and the other had a sample size of 965 subjects [22]. The inconsistency in results may also be due to the use of different measures of ALTs in the studies. To compare our results from previous case–control studies, we used the A-TAC score as a proxy for an ASD diagnosis [17]; rs4307059 was not significantly associated in this case–control analysis (Table 2). Notably, we had low statistical power in these analyses due to the small number of children with A-TAC scores corresponding to an ASD diagnosis.

**Table 2:** **ASD** ^**a**^ **case–control analyses**

Gene	SNP	Genotype	N (case/control)	***P***value ^b^	OR (CI 95%)
*CDH 9/10*	rs4307059	C/C	12/1,651	0.835	1.087 (0.541-2.184)
		T/C	37/5,527		1.160 (0.714-1.884)
		T/T	36/4,730		
*CNTNAP2*	rs2710102	A/A	25/3,223	0.800	0.806 (0.419-1.551)
		A/G	44/5,932		0.846 (0.468-1.529)
		G/G	17/2,735		
*CNTNAP2*	rs7794745	A/A	41/4,963	0.125	0.351 (0.127-0.972)
		T/A	40/5,493		0.422 (0.152-1.168)
		T/T	5/1,559		
*RELN*	rs362691	C/C + C/G	20/2,820	0.933	1.023 (0.601-1.742)
		G/G	63/9,076		
*SHANK3*	rs9616915	C/C	30/3,086	0.094	0.764 (0.425-1.373)
		T/C	31/5,808		1.371 (0.769-2.447)
		T/T	21/2,906		

^a^Above the score 8.5 on A-TAC.

^b^Uncorrected *P* value.

ASD: Autism spectrum disorder; A-TAC: Autism-Tics, ADHD, and Other Co-morbidities inventory.

The rs362691 in *RELN* has been associated with ASD in a recent meta-analysis that included five association studies [24]; one of the included studies showed significant association [25]. In *SHANK3,* mainly rare genetic variations have been identified in subjects with ASD [14] and association analysis for rs9616915 has previously shown negative results for association with ASD in both European and Han Chinese populations [26]. The previous negative findings do not rule out a potential influence of common variation in *SHANK3* or *RELN* on autistic-like traits, which has not been investigated previously. However, in our study we did not find significant associations between ALTs and rs9616915 or rs362691 (Table 1).

Since there is a large co-morbidity between NDPs, we also explored the influence of our ASD candidate SNPs on four genetic NDP factors designed to have 100% heritability [18], that is, we explored the genetic etiology of the co-morbidity among NDPs. These analyses did not show a major influence of these SNPs on the genetic NDP factors (Table 3).

**Table 3:** **Association between genetic factors for neurodevelopmental problems (NDPs) and five SNPs**

			General NDP ^a^						Impulsivity ^a^			Learning problems ^a^			Tics and autism ^a^		
Gene/SNP	MAF	Genotype	N	***A***ll	N	***B***oys	N	***G***irls	***A***ll	***B***oys	***G***irls	***A***ll	***B***oys	***G***irls	***A***ll	***B***oys	***G***irls
*CDH9/10*																	
rs4307059	0.37(C)	C/C	1,149	0.128 (0.70)	538	0.235 (0.72)	611	0.034 (0.66)	0.032 (0.53)	0.034 (0.53)	0.030 (0.52)	0.002 (0.59)	0.017 (0.62)	-0.010 (0.57)	0.067 (0.51)	0.144 (0.51)	-0.002 (0.49)
		T/C	3,923	0.172 (0.71)	1,998	0.275 (0.73)	1,925	0.064 (0.68)	0.028 (0.54)	0.021 (0.55)	0.034 (0.53)	0.051 (0.62)	0.085 (0.65)	0.016 (0.58)	0.069 (0.55)	0.138 (0.58)	-0.003 (0.50)
		T/T	3,424	0.132 (0.69)	1,711	0.244 (0.71)	1,713	0.019 (0.66)	0.023 (0.54)	0.038 (0.55)	0.009 (0.52)	0.031 (0.62)	0.066 (0.65)	-0.004 (0.59)	0.057 (0.50)	0.105 (0.53)	0.009 (0.47)
*P* value^b^				0.962		0.661		0.811	0.554	0.571	0.959	0.401	0.474	0.702	0.734	0.702	0.695
Effect size (CI 95%)^c^				0.001 (-0.021, 0.022)		0.007 (-0.024, 0.037)		-0.004 (-0.034, 0.027)	0.005 (-0.011, 0.021)	0.006 (-0.016, 0.029)	0.001 (-0.024, 0.025)	-0.007 (-0.023, 0.009)	-0.008 (-0.028, 0.013)	-0.005 (-0.033, 0.022)	0.002 (-0.012, 0.017)	0.004 (-0.017, 0.025)	0.004 (-0.018, 0.026)
*CNTNAP2*																	
rs2710102	0.48(G)	A/A	2,316	0.121 (0.70)	1,153	0.203 (0.72)	1,163	0.040 (0.68)	0.004 (0.53)	0.002 (0.54)	0.007 (0.51)	0.050 (0.61)	0.056 (0.62)	0.043 (0.60)	0.057 (0.53)	0.126 (0.56)	-0.012 (0.49)
		A/G	4,242	0.158 (0.70)	2,126	0.274 (0.72)	2,116	0.041 (0.65)	0.030 (0.54)	0.037 (0.56)	0.023 (0.53)	0.033 (0.63)	0.075 (0.67)	-0.010 (0.58)	0.069 (0.52)	0.125 (0.55)	0.013 (0.49)
		G/G	1,924	0.164 (0.70)	966	0.268 (0.71)	958	0.059 (0.68)	0.040 (0.54)	0.036 (0.55)	0.043 (0.53)	0.027 (0.60)	0.063 (0.63)	-0.008 (0.57)	0.066 (0.53)	0.130 (0.56)	0.001 (0.49)
*P* value^b^				0.501		0.135		0.634	0.344	0.315	0.767	0.450	0.692	0.167	0.768	0.856	0.746
Effect size (CI 95%)^c^				0.007 (-0.014, 0.029)		0.025 (-0.008, 0.057)		-0.006 (-0.032, 0.019)	0.008 (-0.009, 0.025)	0.013 (-0.013, 0.039)	0.003 (-0.018, 0.025)	-0.007 (-0.025, 0.011)	0.006 (-0.024, 0.037)	-0.015 (-0.037, 0.006)	0.002 (-0.013, 0.018)	-0.002 (-0.027, 0.022)	0.002 (-0.008, 0.012)
rs7794745	0.36(T)	A/A	3,499	0.144 (0.70)	1,714	0.251 (0.72)	1,785	0.041 (0.67)	0.023 (0.53)	0.028 (0.54)	0.019 (0.53)	0.022 (0.61)	0.048 (0.63)	-0.004 (0.58)	0.075 (0.53)	0.140 (0.55)	0.013 (0.49)
		T/A	3,947	0.156 (0.70)	2,010	0.269 (0.71)	1,937	0.039 (0.67)	0.028 (0.54)	0.035 (0.56)	0.021 (0.53)	0.046 (0.62)	0.082 (0.66)	0.009 (0.58)	0.058 (0.53)	0.116 (0.56)	-0.001 (0.49)
		T/T	1,110	0.146 (0.70)	556	0.225 (0.73)	554	0.068 (0.65)	0.031 (0.53)	0.020 (0.56)	0.042 (0.51)	0.042 (0.63)	0.067 (0.63)	0.017 (0.62)	0.050 (0.51)	0.111 (0.53)	-0.012 (0.48)
*P* value^b^				0.583		0.169		0.583	0.590	0.563	0.905	0.811	0.547	0.296	0.112	0.210	0.567
Effect size (CI 95%)^c^				-0.006 (-0.028, 0.016)		-0.023 (-0.055, 0.010)		0.007 (-0.019, 0.034)	0.005 (-0.012, 0.021)	0.007 (-0.017, 0.031)	0.001 (-0.021, 0.023)	0.002 (-0.014, 0.018)	-0.008 (-0.032, 0.017)	0.012 (-0.01, 0.034)	-0.012 (-0.026, 0.003)	-0.015 (-0.038, 0.008)	-0.003 (-0.013, 0.007)
*RELN*																	
rs362691	0.13(C)	C/C	156	0.097 (0.68)	80	0.149 (0.69)	76	0.043 (0.66)	-0.065 (0.52)	-0.111 (0.45)	-0.017 (0.58)	0.097 (0.68)	0.094 (0.56)	0.100 (0.78)	0.087 (0.51)	0.194 (0.53)	-0.025 (0.46)
		C/G	1,823	0.126 (0.69)	874	0.270 (0.69)	949	-0.007 (0.65)	0.019 (0.55)	0.055 (0.57)	-0.015 (0.53)	0.025 (0.63)	0.055 (0.66)	-0.002 (0.60)	0.063 (0.54)	0.114 (0.58)	0.016 (0.49)
		G/G	6,483	0.156 (0.70)	3,270	0.253 (0.72)	3,213	0.058 (0.67)	0.029 (0.53)	0.023 (0.55)	0.036 (0.52)	0.039 (0.61)	0.071 (0.65)	0.006 (0.57)	0.063 (0.52)	0.128 (0.55)	-0.002 (0.49)
*P* value^b^				0.545		0.879		0.288	0.444	0.856	0.115	0.957	0.802	0.521	0.632	0.715	0.646
Effect size (95% CI)^c^				-0.010 (-0.041, 0.022)		0.003 (-0.04, 0.047)		-0.026 (-0.073, 0.022)	-0.009 (-0.033, 0.014)	0.003 (-0.029, 0.035)	-0.030 (-0.067, 0.007)	0.001 (-0.023, 0.024)	-0.004 (-0.033, 0.026)	0.014 (-0.029, 0.056)	0.005 (-0.016, 0.026)	0.006 (-0.025, 0.036)	0.008 (-0.026, 0.042)
*SHANK3*																	
rs9616915	0.49(T)	C/C	2,170	0.149 (0.71)	1,123	0.263 (0.73)	1,047	0.028 (0.65)	0.039 (0.54)	0.041 (0.56)	0.037 (0.52)	0.020 (0.60)	0.039 (0.62)	0.001 (0.58)	0.060 (0.54)	0.140 (0.55)	-0.025 (0.51)
		C/T	4,182	0.161 (0.70)	2,044	0.266 (0.72)	2,138	0.060 (0.66)	0.037 (0.54)	0.053 (0.55)	0.022 (0.53)	0.039 (0.61)	0.075 (0.64)	0.004 (0.58)	0.057 (0.52)	0.096 (0.54)	0.020 (0.49)
		T/T	2,061	0.120 (0.69)	1,048	0.232 (0.70)	1,013	0.005 (0.66)	-0.010 (0.52)	-0.030 (0.54)	0.011 (0.50)	0.042 (0.63)	0.083 (0.68)	-0.001 (0.57)	0.081 (0.53)	0.169 (0.58)	-0.009 (0.46)
*P* value^b^				0.703		0.870		0.716	0.152	0.068	1.00	0.377	0.308	0.994	0.273	0.266	0.659
Effect size (CI 95%)^c^				0.004 (-0.017, 0.025)		0.002 (-0.027, .032)		0.006 (-0.025, 0.037)	-0.012 (-0.028, 0.004)	-0.020 (-0.042, 0.001)	0.001 (-0.025, 0.025)	0.007 (-0.009, 0.023)	0.011 (-0.01, 0.031)	0.001 (-0.028, 0.028)	0.008 (-0.006, 0.022)	0.012 (-0.009, 0.032)	0.005 (-0.017, 0.028)

^a^Mean values (SD).

^b^Uncorrected *P* value.

^c^Effect size presented as the regression coefficient (95% CI).

A-TAC: Autism-Tics, ADHD, and Other Co-morbidities inventory.

In line with the GWAS published for autistic traits [22, 23], we did not find significant association between the investigated SNPs and ALTs in our large population, although nominal significance is observed for the *CNTNAP2* SNP rs7794745. Hence, our results suggest that these genes, which previously have been found associated with ASD diagnosis, do not have any major influence on ALTs in children from the general population. However, it cannot be excluded that other variants in the same genes may have an effect since our selected SNPs do not capture all variations within our selected genes. Further studies that investigate the influence of genetic variation on different measures of ALTs are highly warranted to better understand the genetics of autism related phenotypes and neurodevelopmental disorders.

## Conclusion

Taken together, our association analyses between the investigated autism candidate regions and ALTs do not suggest a major influence on ALTs in children from the general population.

## Electronic supplementary material

Additional file 1: **Association analyses adjusted for age.** The additional file includes three additional Tables. **Table S1.** Association analyses between autistic-like traits and five SNPs after adjustment for age. **Table S2.** ASD case–control analyses after adjustment for age and **Table S3.** Association analyses between genetic factors for neurodevelopmental problems and five SNPs after adjustment for age. (PDF 153 KB)

## Abbreviations

A-TAC: Autism-Tics, Attention-Deficit/Hyperactivity Disorder, and Other Co- morbidities inventory
ALT: Autistic-like trait
ASD: Autism Spectrum disorder
CATSS: Child and Adolescent Twin Study in Sweden
*CNTNAP2*: *Contactin associated protein-like 2*
*NDP*: *Neurodevelopmental Problem*
*RELN*: *Reelin*
*SHANK3*: *SH3 and multiple ankyrin repeat domains 3*
*SNP*: *Single nucleotide polymorphism*.
