# Supplementary material for: Association study between autistic-like traits and polymorphisms in the autism candidate regions RELN, CNTNAP2, SHANK3, and CDH9/10
Source: Mol Autism. 2014 Dec 16;5:55. doi: 10.1186/2040-2392-5-55 (PMC4276093; doi:10.1186/2040-2392-5-55)
Supplement: Supplementary file 1 — Additional file 1: Association analyses adjusted for age. The additional file includes three additional Tables. Table S1. Association analyses between autistic-like traits and five SNPs after adjustment for age. Table S2. ASD case–control analyses after adjustment for age and Table S3. Association analyses between genetic factors for neurodevelopmental problems and five SNPs after adjustment for age. (PDF 153 KB) [file 13229_2014_146_MOESM1_ESM.pdf]

Additional table 1. Association analyses between autistic-like traits and five SNPs after adjustment for age[a].

**Autistic-like traits**			All			Boys			Girls		
		SNP	p-value	effect size[b]	CI (95%)	p-value	effect size[b]	CI (95%)	p-value	effect size[b]	CI (95%)
	CDH 9/10	rs4307059	0.734	-0.007	0.033, 0.047	0.632	0.016	-0.051, 0.083	0.732	0.007	-0.033, 0.048
	CNTNAP2	rs2710102	0.507	0.013	-0.026, 0.052	0.647	0.015	-0.050, 0.080	0.716	0.008	-0.033, 0.048
	CNTNAP2	rs7794745	0.104	-0.034	-0.075, 0.007	0.045	-0.069	-0.136, -0.002	0.712	0.008	-0.034, 0.050
	RELN	rs362691	0.343	-0.028	-0.086, 0.030	0.628	-0.024	-0.120, 0.072	0.676	-0.013	-0.072, 0.047
	SHANK3	rs9616915	0.196	-0.025	-0.064, 0.013	0.426	-0.026	-0.089, 0.038	0.448	-0.015	-0.055, 0.024
Restricted and Repetitive behavior											
	CDH 9/10	rs4307059	0.159	0.011	-0.004, 0.027	0.302	0.014	-0.013, 0.041	0.164	0.011	-0.005, 0.028
	CNTNAP2	rs2710102	0.583	0.004	-0.011, 0.020	0.565	0.008	-0.018, 0.033	0.888	-0.001	-0.017, 0.015
	CNTNAP2	rs7794745	0.127	-0.012	-0.028, 0.004	0.081	-0.024	-0.051, 0.003	0.871	-0.001	-0.018, 0.015
	RELN	rs362691	0.353	-0.011	-0.034, 0.012	0.738	-0.007	-0.045, 0.032	0.623	-0.006	-0.029, 0.018
	SHANK3	rs9616915	0.184	-0.010	-0.026, 0.005	0.274	-0.014	-0.040, 0.011	0.665	-0.003	-0.019, 0.012
Social interaction impairment											
	CDH 9/10	rs4307059	0.794	-0.002	-0.018, 0.014	0.762	-0.004	-0.030, 0.022	0.673	0.004	-0.014, 0.022
	CNTNAP2	rs2710102	0.833	-0.002	-0.017, 0.014	0.652	-0.006	-0.031, 0.020	0.762	0.003	-0.015, 0.020
	CNTNAP2	rs7794745	0.31	-0.008	-0.025, 0.008	0.050	-0.026	-0.053, <0.01	0.303	0.010	-0.009, 0.028
	RELN	rs362691	0.946	-0.001	-0.024, 0.023	0.834	0.004	-0.034, 0.042	0.975	<0.001	-0.026, 0.026
	SHANK3	rs9616915	0.165	-0.011	-0.027, 0.005	0.185	-0.017	-0.042, 0.008	0.779	-0.002	-0.02, 0.015
Language impairment											
	CDH 9/10	rs4307059	0.732	-0.003	-0.018, 0.013	0.698	0.005	-0.020, 0.030	0.352	-0.008	-0.026, 0.009
	CNTNAP2	rs2710102	0.321	0.008	-0.008, 0.023	0.359	0.012	-0.013, 0.036	0.537	0.006	-0.012, 0.023
	CNTNAP2	rs7794745	0.184	-0.011	-0.027, 0.005	0.292	-0.014	-0.039, 0.012	0.514	-0.006	-0.025, 0.012
	RELN	rs362691	0.289	-0.012	-0.035, 0.011	0.305	-0.019	-0.056, 0.017	0.689	-0.005	-0.032, 0.021
	SHANK3	rs9616915	0.841	-0.002	-0.017, 0.014	0.635	0.006	-0.018, 0.030	0.434	-0.007	-0.024, 0.010

[a]The children's age when their parents answered the A-TAC questionnaire (9 or 12 years old). [b]Effect size presented as the regression coefficient (95% CI). A-TAC=Autism–Tics, ADHD, and Other Co-morbidities inventory.

Additional table 2. ASD[a] case-control analyses after adjustment for age[b].

Gene	SNP	Genotype	N (case/control)	p-value[c]	OR (CI 95%)
CDH 9/10	rs4307059	C/C	12 / 1651	0.836	1.082 (0.593-2.174)
		T/C	37 / 5527		1.159 (0.714-1.883)
		T/T	36 / 4730		
CNTNAP2	rs2710102	A/A	25 / 3223	0.805	0.810 (0.421-1.559)
		A/G	44 / 5932		0.845 (0.467-1.529)
		G/G	17 / 2735		
CNTNAP2	rs7794745	A/A	41 / 4963	0.129	0.356 (0.129-0.979)
		T/A	40 / 5493		0.422 (0.155-1.172)
		T/T	5 / 1559		
RELN	rs362691	C/C+C/G	20 / 2820	0.927	1.025 (0.602-1.745)
		G/G	63 / 9076		
SHANK3	rs9616915	C/C	30 / 3086	0.094	0.761 (0.423-1.369)
		T/C	31 / 5808		1.369 (0.767-2.447)
		T/T	21 / 2906		

[a]Above the score 8.5 on A-TAC. [b]The children's age when their parents answered the A-TAC questionnaire (9 or 12 years old). [c]Uncorrected p-value. ASD=Autism spectrum disorder. A-TAC=Autism–Tics, ADHD, and Other Co-morbidities inventory.

Additional table 3. Association between genetic factors for neurodevelopmental problems (NDPs) and five SNPs after adjustment for age[a].

General NDP			All			Boys			Girls	
	SNP	p-value	effect size[b]	CI (95%)	p-value	effect size[b]	CI (95%)	p-value	effect size[b]	CI (95%)
CDH 9/10	rs4307059	0.961	0.001	-0.021, 0.022	0.660	0.007	-0.024, 0.037	0.811	-0.004	-0.034, 0.027
CNTNAP2	rs2710102	0.479	0.008	-0.014, 0.030	0.126	0.025	-0.007, 0.058	0.643	-0.006	-0.032, 0.020
CNTNAP2	rs7794745	0.591	-0.006	-0.028, 0.016	0.168	-0.023	-0.055, 0.010	0.573	0.008	-0.019, 0.034
RELN	rs362691	0.553	-0.009	-0.041, 0.022	0.869	0.004	-0.040, 0.048	0.291	-0.025	-0.072, 0.022
SHANK3	rs9616915	0.693	0.004	-0.017, 0.025	0.862	0.003	-0.027, 0.032	0.710	0.006	-0.025, 0.037
**Impulsivity**										
CDH 9/10	rs4307059	0.555	0.005	-0.011, 0.021	0.572	0.006	-0.016, 0.028	0.957	0.001	-0.024, 0.025
CNTNAP2	rs2710102	0.371	0.008	-0.009, 0.025	0.340	0.013	-0.013, 0.038	0.781	0.003	-0.019, 0.025
CNTNAP2	rs7794745	0.601	0.004	-0.012, 0.021	0.559	0.007	-0.017, 0.031	0.930	0.001	-0.021, 0.023
RELN	rs362691	0.431	-0.010	-0.033, 0.014	0.870	0.003	-0.029, 0.034	0.113	-0.030	-0.068, 0.007
SHANK3	rs9616915	0.146	-0.012	-0.028, 0.004	0.065	-0.020	-0.042, 0.001	0.987	<0.001	-0.025, 0.025
**Learning problems**										
CDH 9/10	rs4307059	0.403	-0.007	-0.023, 0.009	0.474	-0.008	-0.028, 0.013	0.708	-0.005	-0.033, 0.022
CNTNAP2	rs2710102	0.793	0.002	-0.014, 0.019	0.543	-0.008	-0.032, 0.017	0.275	0.012	-0.010, 0.034
CNTNAP2	rs7794745	0.496	-0.006	-0.024, 0.012	0.630	0.007	-0.023, 0.038	0.173	-0.015	-0.036, 0.007
RELN	rs362691	0.936	0.001	-0.023, 0.025	0.819	-0.003	-0.033, 0.026	0.517	0.014	-0.028, 0.056
SHANK3	rs9616915	0.362	0.008	-0.009, 0.024	0.298	0.011	-0.010, 0.031	0.997	<0.001	-0.028, 0.028
**Tics & autism**										
CDH 9/10	rs4307059	0.732	0.003	-0.012, 0.017	0.702	0.004	-0.017, 0.025	0.690	0.004	-0.018, 0.026
CNTNAP2	rs2710102	0.113	-0.012	-0.026, 0.003	0.21	-0.015	-0.038, 0.008	0.574	-0.003	-0.013, 0.007
CNTNAP2	rs7794745	0.745	0.003	-0.013, 0.018	0.872	-0.002	-0.027, 0.023	0.742	0.002	-0.008, 0.012
RELN	rs362691	0.625	0.005	-0.016, 0.026	0.710	0.006	-0.025, 0.036	0.644	0.008	-0.026, 0.042
SHANK3	rs9616915	0.269	0.008	-0.006, 0.022	0.263	0.012	-0.009, 0.032	0.656	0.005	-0.017, 0.028

[a]The children's age when their parents answered the A-TAC questionnaire (9 or 12 years old). [b]Effect size presented as the regression coefficient (95% CI). A-TAC=Autism–Tics, ADHD, and Other Co-morbidities inventory.
